# The Multitasking Surface Protein of Staphylococcus epidermidis: Accumulation-Associated Protein (Aap)

**DOI:** 10.1128/mBio.01989-21

**Published:** 2021-09-14

**Authors:** Samane Rahmdel, Friedrich Götz

**Affiliations:** a Dept. of Food Hygiene and Quality Control, Shiraz University of Medical Sciences, Shiraz, Iran; b Microbial Genetics, Interfaculty Institute of Microbiology and Infection Medicine Tübingen (IMIT), University of Tübingen, Tübingen, Germany; c Excellence cluster 2124 Controlling Microbes to Fight Infections (CMFI), University of Tübingen, Tübingen, Germany

**Keywords:** *Staphylococcus epidermidis*, accumulation-associated protein, glycan, skin adherence

## Abstract

The stratum corneum is the outermost layer of the epidermis and is thus directly exposed to the environment. It consists mainly of corneocytes, which are keratinocytes in the last stage of differentiation, having neither nuclei nor organelles. However, they retain keratin filaments embedded in filaggrin matrix and possess a lipid envelope which protects the body from desiccation. Despite the desiccated, nutrient-poor, and acidic nature of the skin making it a hostile environment for most microorganisms, this organ is colonized by commensal microbes. Among the classic skin commensals are Propionibacterium acnes and coagulase-negative staphylococci (CoNS) with Staphylococcus epidermidis as a leading species. An as-yet-unanswered question is what enables S. epidermis to colonize skin so successfully. In their recent article, P. D. Fey and his colleagues (P. Roy, A. R. Horswill, and P. D. Fey, mBio 12:e02908-20, 2021, https://doi.org/10.1128/mBio.02908-20) have brought us one step closer to answering this question.

## COMMENTARY

Microorganisms that colonize the human skin as commensals must be physiologically equipped to live on this inhospitable habitat over long periods of time and must also be tolerated by the host immune system. Bacteria that stimulate the immune system too much are hardly found as compatible commensals on the skin in the absence of irritation (1). The first step to skin colonization is, however, a strong binding of bacteria to skin cells. P. D. Fey and colleagues (2) found that in Staphylococcus epidermidis the cell wall-anchored protein Aap mediates strong adherence to glycan moieties on corneocytes, making this protein seemingly responsible for the permanent colonization of the skin by coagulase-negative staphylococci (CoNS).

Aap was first described by the group of G. Peters (3, 4). The team figured out the role of this surface protein in intercellular aggregation and biofilm formation and accordingly named it accumulation-associated protein (Aap). By heterologous expression of Aap on the surface of Lactococcus lactis, Macintosh et al. (5) demonstrated that Aap mediates binding to corneocytes independently of other adhesive proteins. Like most cell wall-anchored surface proteins, Aap is divided into various domains, each with a different function: the amino-terminal signal peptide is followed by the A domain which comprises a variable number of 16-amino-acid repeats followed by a conserved globular L-type lectin subdomain (6). The A domain is followed by the B domain, the proline/glycine-rich region and, at the very carboxy terminus, the cell wall anchor sequence LPXTG. Although the domain responsible for Aap-mediated adherence to corneocytes turned out to be the lectin subdomain of the A region, the ligand of Aap on the surface of the corneocytes had remained unknown until recently (2). The stratum corneum (SC) barrier is rich in glycans, proteoglycans, and corneodesmosome glycoproteins, although the glycan pattern and quantity may change during SC maturation (7). Therefore, Fey and colleagues speculated that glycan ligands could be an ideal target for Aap-mediated adherence of S. epidermidis to corneocytes. Evidence for this assumption came from the following observations:

a) A recombinant A domain was able to block binding of three different S. epidermidis strains to corneocytes, suggesting that the target sites were occupied by the A domain, thus preventing S. epidermidis binding; this also ruled out other surface proteins, teichoic acids, or the polysaccharide intercellular adhesin (PIA) (8, 9) as potential receptors on the surface of the bacterial cells.
b) Knocking out PIA and SdrF (Serine-aspartate repeat protein F) expression had no effect on the S. epidermidis adherence.
c) Antiserum raised against the A or B domain of Aap decreased bacterial binding to corneocytes.
d) Deglycosylation of the corneocytes’ surface with various deglycosylases decreased adhesion to corneocytes.
e) Antibody-mediated blocking of the highly expressed corneocyte proteins loricrin and cytokeratin-10 had no effect on the adherence of S. epidermidis.

The deglycosylation experiments were the highlights of this work because they brought us closer to the target structures. The most common sugar tags on human skin surface proteins are N-linked glycosylation at Asn-X-Ser/Thr motifs and O-linked glycosylation at Ser/Thr residues (10). Treatment with both peptide-*N*-glycosidase (PNGase) and *O*-glycosidase reduced adherence of S. epidermidis by approximately 50%, suggesting that both of these glycan linkages are expressed and serve as binding targets. Pretreatment of corneocytes with glucosidases that remove 5-*N*-acetylneuraminic acid, fucose, and galactose also decreased adherence while removal of *N*-acetylglucosamine and mannose residues did not affect adherence. These results suggest that the binding of S. epidermidis to corneocytes is due to the interaction of Aap’s lectin domain with glycoproteins and possibly glycolipids. This is *per se* not surprising since lectins are carbohydrate binding proteins, i.e., they specifically bind sugar groups that are part of more complex molecules.

Interestingly, however, Fey and colleagues observed that, although not directly involved in ligand binding, the B domain enhanced binding. They speculate that the B domain may contribute to reduce the electrostatic repulsive forces between the bacterial cell surface and the host surface or increase Aap flexibility. An alternative plausible explanation could be that by omission of the B domain, the distance of the lectin domain from the cell wall is shortened so much that the L-lectin domain becomes partly shielded by the peptidoglycan scaffold of the bacterial cell wall and thus cannot capture the glycan structures on the corneocytes’ surface. A similar situation was described for a cell wall-anchored enzyme (a lipase) wherein a gradual shortening of the cell wall-spanning sequence of the enzyme negatively affected its folding to an active conformation and was correlated with a decreasing activity of the cell-bound lipase (11).

Of note, to fully perform its intercellular aggregation of S. epidermidis, the N-terminal region of Aap needs to be processed since it apparently interferes with the Zn^2+^-dependent dimerization of the B domain (12). The processing is carried out by the S. epidermidis specific metalloprotease SpeA (Sep1) (13, 14). Only the resultant truncated Aap isoforms can efficiently promote cellular aggregation and/or biofilm accumulation through B-domain-mediated intercellular adhesion. The inhibition of biofilm formation by the A domain is attributed to its N-terminal region, not to the lectin subunit (2, 13). Together with the report on the aggregation of the colonizing bacterial cells on the skin (15), these findings suggest that the A-lectin domain of Aap mediates adherence to the host ligand. Its cleavage is subsequently required to enable B-domain-mediated cellular aggregation, which in turn increases skin colonization. The downregulation of SepA expression by the global transcription regulator SarA may allow S. epidermidis to modulate the aggregation process in response to the environmental conditions (2).

Bioinformatic analyses revealed that Aap homologous adhesins with a functional lectin-like domain are also present in the other commensal staphylococcal species including S. hominis, S. haemolyticus, S. saprophyticus, and S. capitis. Of note, the Aap-like protein sequences found in S. simulans and S. warneri lacked the A-lectin domain, explaining why these species showed decreased or peripheral adherence to corneocytes (2). This is in agreement with a recent microbiome analysis of the human skin microbiota where samples were taken from the forearm (antecubital fossa) of healthy subjects (16). The most abundant staphylococcal species included S. aureus, S. epidermidis, *S. capitis*, S. hominis, and *S. simulans*, followed by S. saccharolyticus, *S. haemolyticus*, S. pseudintermedius, S. xylosus, and *S. warneri*. Strikingly, S. aureus was almost as abundant as S. epidermidis, highlighting the need for reconsideration of the assumption that the primary habitat of S. aureus is the nasal cavity. S. aureus controls adhesion and clumping through activity of the ArlRS two-component regulatory system and its downstream effector MgrA. Inactivation of the ArlRS-MgrA cascade has been shown to inhibit S. aureus adhesion to a diverse array of host molecules through overexpression of a subset of surface proteins (Ebh, SraP, and SasG). Turning off this cascade contributes to the virulence defect of S. aureus strains and might allow these strains to adapt to skin during colonization where Ebh, SraP, and SasG may act as alternative adhesins (17, 18). The Aap orthologue SasG seems to be the main contributor to adherence to corneocytes, as MgrA-negative mutants showed significant binding to corneocytes compared with the wild-type S. aureus and mutants lacking both *mgrA* and *sasG* (2).

This brings us to the final point, namely, the old question of what distinguishes pathogenic from nonpathogenic staphylococcal species, or rather, what distinguishes S. aureus from S. epidermidis (19). There is no doubt that S. aureus is an aggressive pathogen due to its numerous virulence factors with cell-destructive power. S. epidermidis, on the other hand, is much less virulent, but it is a master of adhesion; there is hardly any implant material to which S. epidermidis has not been found to adhere (20). In this respect, S. epidermidis, although not aggressive, is a persistent commensal. The dangerousness of S. aureus is that it can be both: it can be an aggressive and toxin-producing pathogen that causes acute infections, and on the other hand, S. aureus can mutate to commensal nontoxigenic variants with high expression of adherence factors and increased antibiotic resistance which are less distinguishable from S. epidermidis and may cause chronic infections.

**FIG 1:**
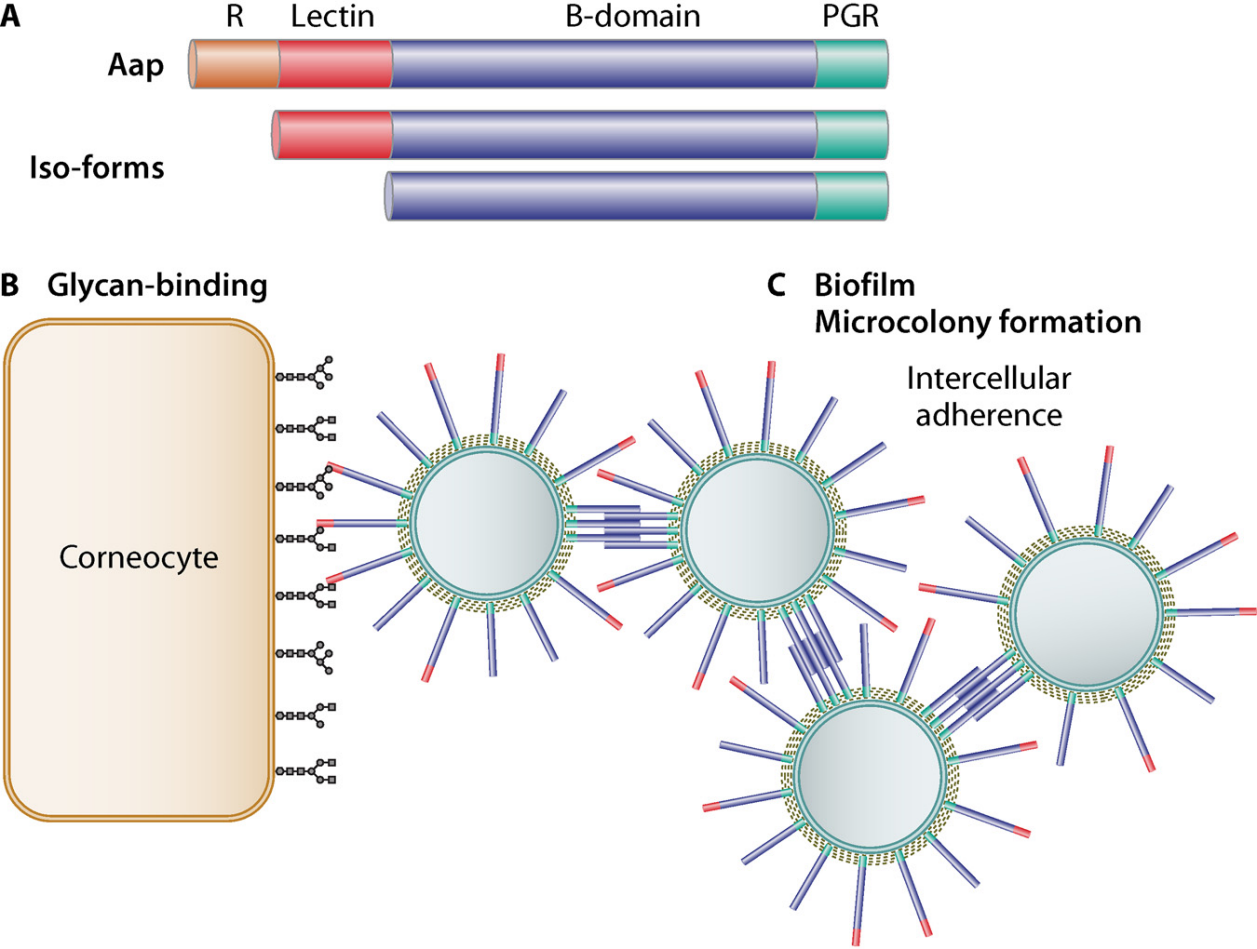
Illustration of the dual functions of Aap. (A) Simplified organization of the cell wall-anchored protein Aap. At the N terminus, it comprises a repeat domain (R) which is followed by the lectin domain, the B domain, the PGR (proline/glycine-rich) region, and the C-terminal LPXTG motif responsible for cell wall anchorage. Aap can be proteolytically truncated into isoforms. (B) The stratum corneum, the outermost layer of skin, is covered by dying or dead cells called corneocytes. Due to the diversity and abundance of transmembrane glycoproteins, proteoglycans, and glycolipids, the corneocyte surface is highly decorated with various glycan moieties which represent the target binding site for Aap. Aap binds to the glycan structures via its lectin subdomain (glycan binding). (C) In the truncated isoforms, the B domain of Aap becomes unhindered and mediates a strong Zn^2+^-dependent intercellular adhesion between S. epidermidis cells, leading to the formation of a biofilm and microcolonies.

In summary, the findings by Roy et al. demonstrate the predominant role of Aap in the colonization of the skin and its corneocytes by S. epidermidis. Aap fulfills two tasks at the same time. The lectin domain mediates binding to glycan structures of skin cells while the truncated isoforms, with their exposed B domain, cause cross-linking between S. epidermidis cells, leading to biofilm and microcolony formation (as illustrated in Fig. 1). We surmise that Aap is a multitasking protein, and its additional functions have yet to be deciphered.
